# Identification and validation of AIB1 and EIF5A2 for noninvasive detection of bladder cancer in urine samples

**DOI:** 10.18632/oncotarget.9406

**Published:** 2016-05-17

**Authors:** Bang-Fen Zhou, Jin-Huan Wei, Zhen-Hua Chen, Pei Dong, Ying-Rong Lai, Yong Fang, Hui-Ming Jiang, Jun Lu, Fang-Jian Zhou, Dan Xie, Jun-Hang Luo, Wei Chen

**Affiliations:** ^1^ Sun Yat-sen University Cancer Center, State Key Laboratory of Oncology in South China, Collaborative Innovation Center for Cancer Medicine, Guangzhou, China; ^2^ Department of Urology, Hainan Provincal Nongken General Hospital, Haikou, Hainan, China; ^3^ Department of Urology, First Affiliated Hospital, Sun Yat-sen University, Guangzhou, China; ^4^ Department of Urology, Sun Yat-sen University Cancer Center, State Key Laboratory of Oncology in South China, Collaborative Innovation Center for Cancer Medicine, Guangzhou, China; ^5^ Department of Pathology, First Affiliated Hospital, Sun Yat-sen University, Guangzhou, China; ^6^ Department of Urology, Meizhou People's Hospital, Guangdong, China

**Keywords:** bladder cancer, AIB1, EIF5A2, diagnostic model, urine biomarkers

## Abstract

We previously demonstrated that amplified in breast cancer 1 (AIB1) and eukaryotic initiation factor 2 (EIF5A2) overexpression was an independent predictor of poor clinical outcomes for patients with bladder cancer (BCa). In this study, we evaluated the usefulness of AIB1 and EIF5A2 alone and in combination with nuclear matrix protein 22 (NMP22) as noninvasive diagnostic tests for BCa. Using urine samples from 135 patients (training set, controls [*n* = 50] and BCa [*n* = 85]), we detected the AIB1, EIF5A2, and NMP22 concentrations using enzyme-linked immunosorbent assay. We applied multivariate logistic regression analysis to build a model based on the three biomarkers for BCa diagnosis. The diagnostic accuracy of the three biomarkers and the model were assessed and compared by the area under the curve (AUC) of the receiver operating characteristic. We validated the diagnostic accuracy of these biomarkers and the model in an independent validation cohort of 210 patients. In the training set, urinary concentrations of AIB1, EIF5A2, and NMP22 were significantly elevated in BCa. The AUCs of AIB1, EIF5A2, NMP22, and the model were 0.846, 0.761, 0.794, and 0.919, respectively. The model had the highest diagnostic accuracy when compared with AIB1, EIF5A2, or NMP22 (*p* < 0.05 for all). The model had 92% sensitivity and 92% specificity. We obtained similar results in the independent validation cohort. AIB1 and EIF5A2 show promise for the noninvasive detection of BCa. The model based on AIB1, EIF5A2, and NMP22 outperformed each of the three individual biomarkers for detecting BCa.

## INTRODUCTION

Bladder cancer (BCa) is responsible for approximately 250,000 deaths per year worldwide and is diagnosed in approximately 900,000 men and women each year [1]. To date, accurate noninvasive detection and monitoring of BCa remains a challenge. At present, several commercially available tests involving urinary biomarkers are available, including nuclear matrix protein 22 (NMP22), signal transducer and activator of transcription (STAT), kinesin-binding trafficking protein (TRAK), ImmunoCyt, BLCA-4, and UroVysion [2]. Of these, only NMP22 has been approved for BCa screening in patients with hematuria. However, none of these assays alone is sufficient for diagnosing or following BCa in clinical practice. Numerous promising single biomarkers for detecting BCa, including carcinoembryonic antigen–related cell adhesion molecule 1 (CEACAM1) [3], tumor-associated trypsin inhibitor (TATI) [4], vascular endothelial growth factor (VEGF) [5], and SERPINA1 [6], have been investigated. The ideal biomarker should be urine-based, sensitive, specific, and cost-effective, but these markers all lack one or more of these qualities.

Considering variation among individual subjects, crosstalk between molecular pathways, and solid tumor heterogeneity, molecular diagnostic panels composed of multiple biomarkers are likely to be far more accurate than single markers. Recently, several studies embraced the multiplex approach and identified the signatures of diagnostic biomarkers. These studies showed that integrating multiple biomarkers into a single model would substantially improve diagnostic value as compared with a single biomarker [7–9]. However, these studies were limited by the lack of independent validation.

Numerous studies have identified amplified in breast cancer 1 (AIB1) [10–12] and eukaryotic initiation factor 2 (EIF5A2) [13–19] as oncogenes. Previously, we showed that AIB1 [20–22] and EIF5A2 [23–25] overexpression was an independent predictor of poor clinical outcomes for patients with BCa.

The aim of this study was to evaluate AIB1 and EIF5A2 as single biomarkers for detecting BCa, and in comparison and in combination with NMP22. We used multivariate binary logistic regression analysis to develop a model integrating AIB1, EIF5A2, and NMP22 to yield a prediction rule. We then assessed the diagnostic accuracy of this model in a training set, and validated it in an independent validation cohort.

## RESULTS

### Characteristics of study subjects

Table 1 presents the clinical and pathological characteristics of the subjects in the training and independent validation sets. The training set comprised 85 urine samples from subjects diagnosed with BCa and from 50 non-cancer controls. There were no significant differences in sex, age, and smoking habits between the patients and the controls in the training set (*p* > 0.05 for all). In the training set, 64.7% of patients had non-muscle invasive BCa (NMIBC) and 43.5% had low-grade disease. The independent validation set was comprised of 134 urine samples from subjects with BCa and from 76 non-cancer controls. As in the training set, there were no significant differences in sex, age, and smoking habits between the patients and the controls (*p* > 0.05 for all). In the independent validation set, 67.2% patients had NMIBC and 41.8% had low-grade disease. There were no significant differences in sex, age, smoking habits, pathological T stage, and grade between the patients in the training and independent validation sets (*p* > 0.05 for all).

**Table 1:** Demographic and clinicopathologic characteristics of training and independent validation study cohorts

Characteristic	Training set			Independent validation set			*P*-value^**^
	Non-cancer	Cancer	*P*-value^*^	Non-cancer	Cancer	*P*-value^*^	
**Total (n)**	50	85		76	134		
**Sex (n)**			0.905^a^			0.549^a^	
Male	41 (82.0%)	69 (81.2%)		58 (76.3%)	107 (79.9%)		0.810^a^
Female	9 (18.0%)	16 (18.8%)		18 (23.7%)	27 (20.1%)		
**Median age (IQR; years)**	61 (14)	64 (18)	0.230^b^	60 (19)	62 (18)	0.346^b^	0.271^b^
**Smoking habits**	27 (54.0%)	49 (57.6%)	0.680^a^	44 (57.9%)	70 (52.2%)	0.429^a^	0.434^a^
**Pathologic T stage**	Not applicable			Not applicable			
NMIBC (pTa, pTis, pT1)		55 (64.7%)			90 (67.2%)		0.708^a^
MIBC (pT2-pT4)		30 (35.3%)			44 (32.8%)		
**Grade**	Not applicable			Not applicable			
Low grade		37 (43.5%)			56 (41.8%)		0.800^a^
High grade		48 (56.5%)			78 (58.2%)		

IQR = interquartile range; NMIBC = non-muscle-invasive bladder cancer; MIBC = muscle-invasive bladder cancer;

a Chi-square test

b Mann-Whitney U test.

* Comparison of the non-cancer group by the cancer group in the same set.

** Comparison of the cancer group in the taring set by the cancer group in the independent set.

### Assessment of repeatability of enzyme-linked immunosorbent assay (ELISA) data

**Figure 1:**
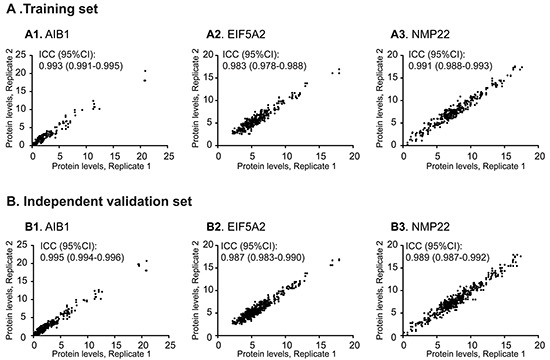
Repeatable validation of measurements of urinary AIB1, EIF5A2, and NMP22 levels using ELISA Highly concordant results between replicate measurements of AIB1 (**A1**, **B1**), EIF5A2 (**A2**, **B2**), and NMP22 (**A3**, **B3**) were obtained in the training and independent validation sets.

Initially, we aimed to verify that protein measurements could be reliably obtained from urine samples. For this purpose, replicate measurements of AIB1, EIF5A2, and NMP22 in urine samples from the training and independent validation sets were performed. Scatter plots were constructed to display the repeatability of three consecutive measurements across all urine samples from the training and independent validation sets. In this study, all intraclass correlation coefficients (ICCs) were >0.98, indicating that repeatability was reliable across the entire cohort (Figure 1). Given these results, we concluded that our ELISA workflow was well suited to reliably determining urinary AIB1, EIF5A2, and NMP22 levels.

### Association of urinary AIB1, EIF5A2, and NMP22 levels with BCa presence and patient characteristics

**Figure 2:**
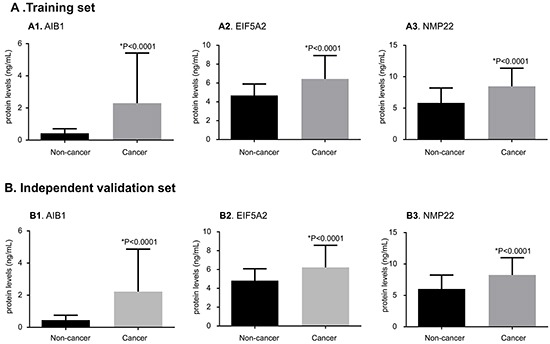
Comparisons of urinary AIB1, EIF5A2, and NMP22 levels between BCa and non-cancer groups in the training and independent validation sets The protein levels of AIB1 (**A1**, **B1**), EIF5A2 (**A2**, **B2**), and NMP22 (**A3**, **B3**) are substantially elevated in BCa urine samples when compared with the non-cancer urine samples in the training and independent validation sets (*p <* 0.0001 for both). The results in all groups are presented as the mean and SD. Significance (*p* < 0.05) was assessed using the Mann–Whitney U test.

Figure 2 shows the urinary concentrations of the three proteins of the training and independent validation sets. In the training set, the mean urinary levels of AIB1 (1.40 vs. 0.35 ng/ml), EIF5A2 (5.83 vs. 4.56 ng/ml), and NMP22 (7.74 vs. 6.31 ng/ml, each *p* < 0.0001) were significantly higher in the subjects with BCa than in the controls. In the independent validation set, the mean urinary levels of AIB1 (1.31 vs. 0.39 ng/ml), EIF5A2 (5.74 vs. 4.60 ng/ml), and NMP22 (7.78 vs. 6.23 ng/ml, each *p* < 0.0001) were significantly higher in the subjects with BCa than in the controls.

The association of urinary AIB1, EIF5A2, and NMP22 levels with presence of BCa and patient characteristics is shown in Table 2. Among the patients with BCa in the training set, urinary concentrations of AIB1, EIF5A2, and NMP22 were significantly elevated in patients with MIBC compared to those with NMIBC (*p* < 0.05 for all) and in patients with high-grade disease as compared to patients with low-grade disease (*p* < 0.05 for all). Interestingly, we obtained similar results in the independent validation set (Table 2). However, all of the investigated tumor marker levels did not differ significantly among the BCa and non-cancer control groups as classified by sex, age, or smoking habits in the training and independent validation sets (*p* > 0.05 for all; Table 2).

**Table 2:** Association of urinary levels of the three markers with selected patient characteristics

	Training set (n=135)				Independent validation set (n=210)			
	Non-cancer (n=50)		Cancer (n=85)		Non-cancer (n=76)		Cancer (n=134)	
	Median (IQR)	*P*-value^*^	Median (IQR)	*P*-value^*^	Median (IQR)	*P*-value^*^	Median (IQR)	*P*-value^*^
**AIB1 (ng/ml)**								
**Sex**								
Male	0.35(0.16)	0.216	1.31(2.14)	0.372	0.36(0.17)	0.170	1.21(1.82)	0.286
Female	0.29(0.28)		1.46(1.58)		0.42(0.19)		1.65(2.74)	
**Age (years)**								
≥65	0.34(0.21)	0.291	1.52(1.70)	0.657	0.43(0.33)	0.276	1.57(2.07)	0.775
<65	0.36(0.17)		1.32(2.29)		0.37(0.14)		1.10(2.22)	
**Smoking habits**								
Yes	0.34(0.19)	0.683	1.47(2.19)	0.445	0.39(0.21)	0.739	1.52(2.05)	0.484
No	0.37(0.20)		1.36(1.76)		0.35(0.18)		1.26(2.36)	
**Clinical stage**								
NMIBC			0.69(1.52)	<0.0001			0.67(1.56)	<0.0001
MIBC			2.13(3.12)				2.23(3.52)	
**Grade**								
Low			0.74(1.59)	0.030			0.64(1.36)	<0.0001
High			1.62(2.41)				1.71(2.58)	
**EIF5A2 (ng/ml)**								
**Sex**								
Male	4.65 (0.99)	0.251	5.82(2.02)	0.706	4.61(0.98)	0.287	5.74(2.43)	0.517
Female	4.21(1.69)		6.18(3.72)		4.26(1.60)		5.54(1.75)	
**Age (years)**								
≥65	4.81(0.81)	0.190	5.97(2.15)	0.175	4.76(1.40)	0.211	5.91(1.85)	0.201
<65	4.15(1.00)		5.48(2.94)		4.34(1.19)		5.45(2.64)	
**Smoking**								
Yes	4.61(1.10)	0.419	6.00(2.19)	0.435	4.59(1.00)	0.600	5.89(2.26)	0.362
No	4.40(1.00)		5.70(2.46)		4.36(1.13)		5.63(2.55)	
**Clinical stage**								
NMIBC			5.63(1.91)	0.006			5.57(1.74)	<0.0001
MIBC			6.75(4.62)				6.77(4.83)	
**Grade**								
Low			5.52(1.24)	0.002			5.49(1.18)	<0.0001
High			6.55(3.01)				6.34(3.03)	
**NMP22 (ng/ml)**								
**Sex**								
Male	6.41(2.02)	0.109	7.87(2.68)	0.131	6.37(2.06)	0.096	7.79(2.39)	0.153
Female	4.61(3.41)		7.14(2.55)		5.01(4.08)		6.98(4.84)	
**Age (years)**								
≥65	6.28(2.34)	0.446	7.79(3.26)	0.404	6.38(2.61)	0.467	7.97(2.16)	0.594
<65	6.56(2.09)		7.63(2.28)		6.09(2.16)		7.70(2.83)	
**Smoking**								
Yes	6.51(2.24)	0.459	7.81(2.64)	0.456	6.41(2.07)	0.493	8.05(2.91)	0.424
No	6.11(2.51)		7.37(3.14)		6.23(3.20)		7.67(2.19)	
**Clinical stage**								
NMIBC			7.37(2.28)	0.001			7.56(2.08)	<0.0001
MIBC			8.56(4.46)				8.92(5.57)	
**Grade**								
Low			7.05(1.29)	<0.0001			7.04(1.53)	<0.0001
High			9.19(3.70)				9.03(3.37)	

IQR = interquartile range; NMIBC = non-muscle-invasive bladder cancer; MIBC = muscle-invasive bladder cancer.

* Mann-Whitney U test.

### Diagnostic performance of urinary AIB1, EIF5A2, and NMP22 levels for predicting BCa

**Figure 3:**
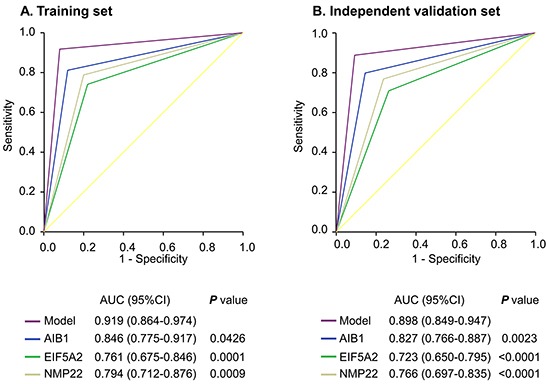
ROC curves comparing the diagnostic performance of the combination model with individual AIB1, EIF5A2, or NMP22 in the training and independent validation sets Comparisons of the diagnostic performance by the model and individual AIB1, EIF5A2 or NMP22 in the training cohort **A.** and independent validation set **B.**.

The cut-off values for AIB1, EIF5A2, and NMP22 were 0.58 ng/ml, 5.06 ng/ml, and 6.89 ng/ml, respectively. Figure 3A demonstrates the receiver operating characteristic (ROC) curves of the AIB1, EIF5A2, and NMP22 assays in the training set. Table 3 shows the urinary biomarker diagnostic rates. Urinary AIB1 had 81% sensitivity, 88% specificity, and an area under the ROC curve (AUC) of 0.846 (95% confidence interval [95% CI] 0.775–0.917). Urinary EIF5A2 had 74% sensitivity, 78% specificity, and an AUC of 0.761 (95% CI 0.675–0.846). Urinary NMP22 had 79% sensitivity, 80% specificity, and an AUC of 0.794 (95% CI 0.712–0.876).

**Table 3:** Urinary biomarker diagnostic rates yield in the training and independent validation sets

	AIB1	EIF5A2	NMP22	Model
**Training set**				
Cutoff value (ng/ml)	0.58	5.06	6.89	10.08
Sensitivity %(95% CI)	81(71-89)	74(64-83)	79(69-87)	92(84-97)
Specificity %(95% CI)	88(76-96)	78(64-89)	80(66-90)	92(81-98)
PPV %(95% CI)	92(84-97)	85(75-92)	87(77-94)	95(88-99)
NPV %(95% CI)	73(60-84)	64(51-76)	69(56-81)	87(75-95)
**Independent validation set**				
Sensitivity %(95% CI)	80(72-86)	71(62-78)	77(69-84)	89(82-94)
Specificity %(95% CI)	86(76-93)	74(62-83)	76(65-85)	91(82-96)
PPV %(95% CI)	91(84-95)	83(74-89)	85(78-91)	94(89-98)
NPV %(95% CI)	71(60-80)	59(48-69)	65(54-75)	82(72-90)

CI = confidence interval; PPV= positive predictive value; NPV= negative predictive value

To confirm that the three markers had similar diagnostic performances in different populations, we validated them in the independent set of 210 patients. We applied the cut-off values yielded in the training set to the independent validation set. Interestingly, the results were similar (Figure 3B; Table 3).

We compared the AIB1 or EIF5A2 AUCs with that of NMP22, and found that they were not statistically different for both comparisons in the training set (*p* > 0.05 for all). However, in the independent validation cohort, the AIB1 AUC was significantly greater than that of NMP22 (*p* = 0.0264).

The ability of urinary AIB1 and EIF5A2 to predict patients with NMIBC was analyzed using nonparametic ROC analysis. In the trainng set, AUCs for urinary AIB1 and EIF5A2 were 0.804 (95% CI 0.715–0.875) and 0.708 (95% CI 0.611–0.793), respectively. Urinary AIB1 had 73% (95% CI 59%–84%) sensitivity, 88% (95% CI 76%–96%) specificity. Urinary EIF5A2 had 64% (95% CI 50%–76%) sensitivity, 78% (95% CI 64%–89%) specificity. In the independent validation set, Urinary AIB1 had 73% (95% CI 63%–82%) sensitivity, 86% (95% CI 76%–93%) specificity, and an AUC of 0.792 (95% CI 0.722–0.850). Urinary EIF5A2 had 63% (95% CI 52%–73%) sensitivity, 74% (95% CI 62%–83%) specificity, and an AUC of 0.684 (95% CI 0.608–0.753). (Supplementary data Table).

### Logistic regression analyses of urinary AIB1, EIF5A2, and NMP22 levels for predicting BCa

In univariable logistic regression analyses for the training set, increased AIB1, EIF5A2, and NMP22 were all associated with increased risk of BCa (*p* < 0.0001 for all; Table 4). We performed the same analyses for the independent validation set, and found that higher levels of AIB1, EIF5A2, and NMP22 were associated with increased risk of BCa (*p* < 0.0001 for all; Table 4).

**Table 4:** Logistic regression analysis of biomarkers in voided urine of the training and independent validation sets

Characteristic	Training set			Independent validation set		
	Odds ratio	95% CI	*P*-value	Odds ratio	95% CI	*P*-value
**Univariate analysisa^a^**						
AIB1	31.63	11.50-86.97	<0.0001	23.41	10.89-50.37	<0.0001
EIF5A2	10.15	4.44-23.21	<0.0001	6.82	3.63-12.83	<0.0001
NMP22	14.89	6.26-35.42	<0.0001	10.71	5.51-20.80	<0.0001
Model	128.14	35.58-461.52	<0.0001	78.20	30.40-201.17	<0.0001
**Multivariate analysis**^b^						
AIB1	34.73	12.03-100.22	<0.0001	23.13	10.71-49.95	<0.0001
EIF5A2	11.13	4.75-26.11	<0.0001	6.96	3.67-13.21	<0.0001
NMP22	16.24	6.68-39.53	<0.0001	10.53	5.38-20.58	<0.0001
Model	146.26	36.79-581.50	<0.0001	91.71	33.26-252.88	<0.0001

a Univariate logistic regression analysis;

b Multivariate logistic regression analysis.

In multivariable logistic regression that adjusted for the effects of age, sex, and smoking habits, higher AIB1, EIF5A2, and NMP22 levels in the training set were associated with increased risk of BCa (*p* < 0.0001 for all; Table 4). We applied the same analyses to the independent validation set, and obtained largely the same results (*p* < 0.0001 for all; Table 4).

### Constructing a model combining AIB1, EIF5A2, and NMP22 detection

**Figure 4:**
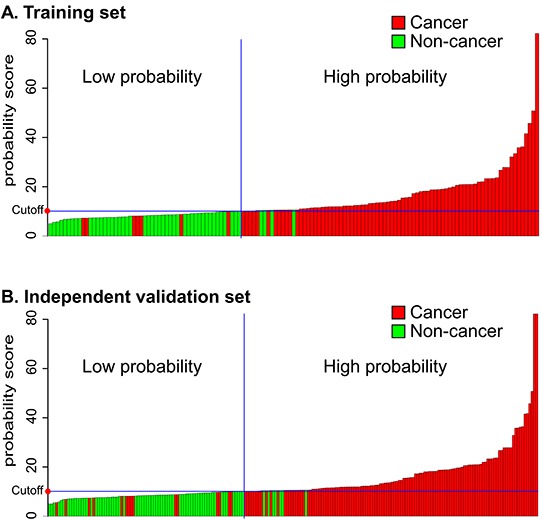
Probability score by combination model in the training and independent validation sets **A.** Training cohort. **B.** Independent validation set. Every column in the diagrams of the two cohorts represents an individual patient.

We constructed a model combining AIB1, EIF5A2, and NMP22 to derive a prediction rule in the training set. We then used the prediction rule to calculate a probability score for every subject based on their individual expression levels of the three biomarkers, where probability score = (3.304 × expression value of AIB1) + (0.828 × expression value of EIF5A2) + (0.546 × expression value of NMP22). The cut-off probability score in the combination detection assay was 10.08. The distribution of probability scores revealed that the combination assay had better diagnostic accuracy (Figure 4A). The combination assay had 92% (95% CI 84%–97%) sensitivity and 92% (95% CI 81%–98%) specificity (Table 3), and yielded an AUC of 0.919 (95% CI 0.864–0.974), outperforming any single biomarker (Figure 3A). In univariable logistic regression analysis, higher urinary biomarker levels in the combination assay were associated with increased risk of BCa presence (Odds Ratio [OR]: 128.14, 95% CI 35.58-461.52, *p* < 0.0001; Table 4). In multivariate logistic regression analysis that adjusted for the effects of age, sex, and smoking habits, the combination assay was associated with increased risk of BCa (OR: 146.26, 95% CI 36.79-581.50, *p* < 0.0001; Table 4).

To confirm that the combination assay had similar diagnostic value in different populations, we applied it to the independent validation set comprising 134 patients from another center and the 76 control subjects. The combination assay yielded similar diagnostic performances, as well as in the training set (Figure 4B). Figure 3B shows the combination assay ROC curve. The sensitivity and specificity were 89% (95% CI 82%–94%) and 91% (95% CI 82%–96%), respectively (Table 3). The combination assay had an AUC of 0.898 (95% CI 0.849–0.947) for BCa detection, outperformed any single biomarker (Figure 3B). In univariable logistic regression analysis, higher urinary biomarker levels in the combination assay were associated with BCa (OR: 78.20, 95% CI 30.40-201.17, *p* < 0.0001; Table 4). In multivariable logistic regression that adjusted for the effects of age, sex, and smoking habits, higher urinary biomarker levels in the combination assay were associated with BCa (OR: 91.71, 95% CI 33.26-252.88, *p* < 0.0001; Table 4).

We used ROC to analyze the ability of the combination assay for prediction of patients with NMIBC. In the training cohort, the combination assay had an AUC of 0.896 (95% CI 0.822–0.947) for NMIBC detection, outperformed both of the urinary AIBI (*p* = 0.02) and EIF5A2 (*p* < 0.0001). The combination assay had 87% (95% CI 76%–95%) sensitivity and 92% (95% CI 81%–98%) specificity. In the independent validation cohort, AUC of the combination assay was 0.872 (95% CI 0.812–0.919), diagnostic performance of the combination assay was significantly greater than both of the urinary AIB1 (*p* = 0.0001) and EIF5A2 (*p* < 0.0001). The combination assay had 84% (95% CI 75%–91%) sensitivity and 91% (95% CI 82%–96%) specificity. (Supplementary data Table).

## DISCUSSION

Our previous studies confirmed that both AIB1 and EIF5A2 are upregulated in BCa tissue. These findings led us to hypothesize that AIB1 and EIF5A2 may be detectable in the urine of patients with BCa. For an assay to be clinically useful, it needs to be performed on a high-throughput platform such as an ELISA. We constructed an ELISA assay for AIB1, EIF5A2, and NMP22. Our results indicate that the repeatability of urinary protein measurements for AIB1, EIF5A2, or NMP22 is extremely high.

In this study, we have demonstrated that increased urinary levels of AIB1, EIF5A2, and NMP22 are significantly associated with the presence of BCa. Moreover, patients with MIBC had higher AIB1, EIF5A2, and NMP22 levels than those with NMIBC. The urinary levels of all three biomarkers were higher in patients with high-grade disease than in those with low-grade disease. Most importantly, urinary AIB1, EIF5A2, and NMP22 levels were all independently associated with BCa. We noted that urinary AIB1 had better diagnostic performance than urinary NMP22 for detecting BCa. To our knowledge, preliminary studies have reported numerous promising single protein biomarkers, such as NMP-9 [26], chemokine ligand 18 (CCL18) [8], BLCA-1 [27], and SERPINA1 [6]. Tilki et al. monitored CEACAM1 as a biomarker for detecting the presence of BCa in voided urine samples from 175 patients (93 cancer cases). The sensitivity and specificity of this biomarker was 74% and 95%, respectively [3]. In a cohort of 160 patients (80 cancer cases), Gkialas et al. showed that TATI had 85.7% sensitivity and 77.5% specificity for diagnosing BCa [4]. Another group reported that VEGF ELISA detected BCa with 83% sensitivity and 87% specificity in a set of 127 patients (64 cancer cases) [5]. However, no sample size in these studies exceeded 200 patients. More importantly, these studies were all limited by the absence of validation. Our study involved 345 patients, including an independent validation cohort of >200 patients.

Detecting BCa using diagnostic markers remains a challenge. The inadequate power of single markers may partly explain this. Combinatorial analysis revealed that the 3-biomarker signature composed of AIB1, EIF5A2, and NMP22 clearly outperformed the individual biomarkers as target proteins for detecting BCa via urinalysis. We found that the 3-biomarker signature achieved high sensitivity, specificity, positive predictive value (PPV), and negative predictive value (NPV) in the two sample sets. As a standalone assay, the 3-biomarker panel had an AUC of 0.919 for BCa detection. Similar results were obtained for the independent validation cohort. Several studies have shown that multiple protein biomarkers could increase the diagnostic performance to higher levels. For example, in a cohort of 127 patients (64 tumor-bearing subjects), Goodison et al. demonstrated that a panel based on two biomarkers (VEGF and apolipoprotein E [APOE]) had 81% sensitivity and 97% specificity for detecting BCa. By combining three biomarkers (interleukin-8 [IL-8], VEGF, and APOE), a logistic prediction model was derived that had 90% sensitivity and 97% specificity [7]. Furthermore, in a set of 127 patients (64 cancer cases), Urquidi et al. identified a predictive model comprised of three biomarkers (CCL18, plasminogen activator inhibitor-1 [PAI-1], and CD44), where the sensitivity and specificity of this model were 86% and 89%, respectively [8]. However, these studies were all limited by the absence of a validation cohort. Recently, Margel et al. built a model using heat shock protein 60 (HSP60) and IL-13 and reported 76% sensitivity and 74% specificity after applying it to an internal validation cohort of 40 patients from the same medical center [9], but the cohort was not an independent validation set. It seems more reasonable that our study includes an independent validation set of 210 patients.

Previous reports have implicated AIB1 in bladder tumor biology, and a few studies have investigated its use as a potential biomarker of BCa [10–12]. Previously, we demonstrated that AIB1 overexpression is correlated with cancer progression after transurethral resection of bladder tumor (TUR-Bt) for BCa [20, 21], and Zhang et al. confirmed that AIB1 was significantly upregulated in BCa tissues [28]. Our further studies demonstrated that AIB1 knockdown mediated by ACC/CaIP6/siRNA complex transfection resulted in inhibited BCa cell proliferation *in vitro* and *in vivo* [22]. We also found that AIB1 induced BCa cell cycle progression via phosphoinositide 3-kinase (PI3K)/AKT and E2F transcription factor 1 (E2F1) to promote proliferation [21]. These findings provide a basis for the concept that AIB1 may serve as a promising biomarker for diagnosing patients with early-stage BCa. In this study, we found that AIB1 can be detected in human urine. In addition, we found that urinary AIB1 had a higher diagnostic accuracy for detecting BCa.

EIF5A2 is overexpressed in many human malignancies and is critically associated with tumor progression, lymph node metastasis, and poor prognosis [14–19, 23]. Our previous studies suggested that EIF5A2 overexpression, as detected by immunohistochemistry, may predict tumor recurrence and progression in patients with pTa/pT1 BCa, and further studies demonstrated that EIF5A2 overexpression is critically correlated with shortened survival of patients with BCa who are treated with radical cystectomy [23, 25]. Our group recently determined that upregulated expression of EIF5A2 in localized invasive BCa is an independent predictor of poor metastasis-free survival. Further study of the molecular mechanisms demonstrated that EIF5A2 increases transforming growth factor-β1 (TGF-β1) expression through STAT3 to induce epithelial–mesenchymal transition and promotes aggressiveness in BCa [24]. In the present study, the ELISA data confirmed that high levels of EIF5A2 were present in the urine samples from the subjects with BCa. It can serve as a useful biomarker for detecting BCa.

The present study is limited because the study was performed in a southern Chinese population from two clinical centers, and the distribution of clinical characteristics might differ in other regions, rendering it susceptible to the inherent biases of such a study format. Therefore, our results should be further validated by multicenter clinical trials and patients from different areas and races.

In summary, our findings show that higher levels of urinary AIB1 and EIF5A2 are associated with the presence of BCa. Moreover, our study demonstrates that the model integrating urinary AIB1, EIF5A2, and NMP22 detection can be a useful tool for diagnosing patients with BCa.

## MATERIALS AND METHODS

### Specimen and data collection

The study was performed after we had obtained approval from the local institutional review board. Informed consent was obtained from each subject. Voided urine samples from 345 subjects were collected prospectively in the morning and frozen at −20°C within 30 min until analyzed. For the training set, urine samples were obtained from 85 patients from the First Affiliated Hospital of Sun Yat-sen University, Guangzhou, China, between November 25, 2013, and October 28, 2014. Fifty control subjects, comprising six patients with renal calculi, five with benign bladder lesions, seven with benign prostatic enlargement, seven with hematuria, six with urinary tract infection, and 19 healthy volunteers, were enrolled. The five patients with benign bladder lesions and the seven patients with benign prostatic enlargement had undergone TUR-Bt and transurethral resection of the prostate, respectively. Another 134 urine samples were obtained from the Cancer Center of Sun Yat-sen University, Guangzhou, China, between November 1, 2013, and November 28, 2014, and from 76 control subjects, comprising seven patients with renal calculi, eight with benign bladder lesions, 10 with benign prostatic enlargement, nine with hematuria, eight with urinary tract infection, and 34 healthy volunteers were enrolled in the independent validation set. The patients with benign bladder lesions and the patients with benign prostatic enlargement had also undergone TUR-Bt and transurethral resection of the prostate, respectively. The histological grade and stage were recorded according to the 2004 World Health Organization grading system and the sixth edition of the tumor-nodes-metastasis (TNM) classification system, respectively. All subjects were confirmed without any symptom of kidney failure. Office cystoscopy was performed in all control subjects with hematuria. According to the International Consensus Panel on Bladder Tumor Markers [29], this cohort would serve as a phase II (validation) study. Data are reported using Standards for Reporting of Diagnostic Accuracy (STARD) criteria [30].

### Urinary sample preparation

Prior to any therapeutic intervention, approximately 50 ml clean-catch, first-morning midstream-voided urine was collected prospectively from each subject. The sample was immediately centrifuged at 1000 ×*g* 4°C for 10 min. Supernatant was frozen at −20°C until analyzed.

### Reagents

ELISA kits of human AIB1 (SEE798Hu) and human NMP22 (NUMA1: SEC332Hu) were purchased from USCN Life Science (Wuhan, China). ELISA paired antibody (H00056648-AP11) and recombinant protein (H00056648-P01) for detecting human urinary EIF5A2 were purchased from Abnova (Taipei, Taiwan). Secondary streptavidin–horseradish peroxidase-conjugated anti-mouse immunoglobulin G antibody (ab6789) was purchased from Abcam (Cambridge, MA, USA).

### Urinary AIB1 and NMP22 measurements

An ELISA kit was used to quantify the urinary AIB1 and NMP22 concentrations. Assays were performed in triplicate according to the manufacturer's instructions. The minimum detection concentrations were 0.124 ng/ml for AIB1 and 0.063 ng/ml for NMP22.

### Urinary EIF5A2 measurements

Sandwich ELISA was used to quantify the urinary EIF5A2 concentrations. Assays were performed in triplicate according to the manufacturer's instructions. The minimum detection concentration was 3 ng/ml.

### Statistical analysis

The association between categorical data and differences of categorical data between the cases and controls were tested using the chi-square test. The nonparametric Mann–Whitney sum rank U test was used for comparing the variables between groups. The precision of the repeated measurements was determined using ICC.

We investigated the diagnostic performance of the three biomarkers for BCa detection. The US National Cancer Institute recommends evaluating the performance of potential markers for cancer detection using ROC curves [31]. Accordingly, we used ROC curve analysis to select a cut-off value defined by the Youden index [32]. Subjects with values ≥ cut-off value were defined as positive (carcinoma detected); subjects with values < cut-off value were defined as negative. Accordingly, we generated nonparametric ROC curves that plotted the outcome (positive or negative) for sensitivity against the false-positive rate (1-specificity) to calculate the sensitivity, specificity, positive predictive value (PPV), negative predictive value (NPV), AUC, and the 95% CI for association with the presence of BCa.

We used multivariate binary logistic regression analysis to construct a model combining AIB1, EIF5A2, and NMP22 to derive a prediction rule in the training set. We determined the Youden index cut-off probability score to maximize the sum of sensitivity, specificity, the PPV and NPV for the combination assay (all three biomarkers in the diagnostic panel). We applied the model to the independent validation cohort. We generated nonparametric ROC curves for the model using the same method described above. We used univariable and multivariable binary logistic regression to calculate odds ratios and 95% CI. Statistical significance in this study was set at *p* < 0.05 and all reported *p*-values were 2-sided. Statistical analyses were done using SPSS 12.0 (SPSS Inc., Chicago, IL, USA) and using MedCalc version 8.0 (MedCalc Software) for ROC curve analysis.

## SUPPLEMENTARY TABLES


