# Supplementary material for: Identification and validation of AIB1 and EIF5A2 for noninvasive detection of bladder cancer in urine samples
Source: Oncotarget. 2016 May 17;7(27):41703–14. doi: 10.18632/oncotarget.9406 (PMC5173089; doi:10.18632/oncotarget.9406)
Supplement: Supplementary file 1 [file oncotarget-07-41703-s001.pdf]

SUPPLEMENTARY TABLE

Supplementary Table S1: Diagnostic efficiency of AIB1 and EIF5A2 alone and model for NMIBC in training and independent validation sets.

	Training set				
	**AIB1**	***P*-value***	**EIF5A2**	***P*-value****	**Model**
AUC (95% CI)	0.804(0.715-0.875)	0.02	0.708(0.611-0.793)	<0.0001	0.896(0.822-0.947)
Sensitivity %(95% CI)	73(59-84)		64(50-76)		87(76-95)
Specificity %(95%CI)	88(76-96)		78(64-89)		92(81-98)
PPV %(95% CI)	87(74-95)		76(61-87)		92(82-98)
NPV %(95% CI)	75(62-85)		66(53-78)		87(75-95)
	**Independent validation set**				
AUC (95% CI)	0.792(0.722-0.850)	0.0001	0.684(0.608-0.753)	<0.0001	0.872(0.812-0.919)
Sensitivity %(95% CI)	73(63-82)		63(52-73)		84(75-91)
Specificity %(95%CI)	86(76-93)		74(62-83)		91(82-96)
PPV %(95% CI)	86(63-93)		74(63-84)		92(84-97)
NPV %(95% CI)	72(62-81)		62(51-72)		82(72-90)

NMIBC=non-muscle-invasive bladder cancer; AUC=area under the curve
P-value* = Model AUC vs AIB1 AUC for the training and independent validation sets
P-value**=Model AUC vs EIF5A2 AUC for the training and independent validation sets
